# Endodontic retreatment decision‐making: The influence of the framing effect

**DOI:** 10.1002/cre2.715

**Published:** 2023-01-29

**Authors:** Thomas Kvist, Daniela Henelius, Agnesa Smakiqi

**Affiliations:** ^1^ Department of Endodontology, Institute of Odontology, Sahlgrenska Academy University of Gothenburg Gothenburg Sweden

**Keywords:** endodontic retreatment decision‐making, informed consent, root filled teeth

## Abstract

**Objective:**

The aim of this study was to explore the influence of a framing effect in retreatment decision‐making of a root‐filled tooth with asymptomatic apical periodontitis (AP).

**Method:**

Two variants of a questionnaire were created in which the factual information about a root filled with AP was identical. The options in the first variant were framed in favor to wait and see (FW) and those in the other variant were framed in favor of retreatment (FR) Two hundred and forty‐eight volunteers were by chance given one of the two versions and were asked to choose between having retreatment or to wait and see.

**Results:**

Of the 125 participants given the FW version, 69 (55.2%) chose to wait and see while out of the 123 participants who were given the FR version, 38 (30.9%) made the alike choice (*p* = .0002).

**Conclusion:**

A framing effect is likely to play an essential role in endodontic retreatment decision‐making of root‐filled teeth with asymptomatic apical periodontitis.

## 1 INTRODUCTION

Epidemiological studies have reported a prevalence of periapical radiolucencies in root‐filled teeth of between 12% and 72% (Kielbassa et al., 2017; Pak et al., 2012; Silnovic et al., 2022). Although diagnosis is not always straightforward, most cases are caused by an inflammatory lesion, apical periodontitis (AP). As AP in root‐filled teeth tends to remain more or less asymptomatic over many years, its first diagnosis is often made during a routine examination or as an incidental finding.

According to the prevailing academic paradigm, a lesion diagnosed as AP in association with a root‐filled tooth is defined as an “endodontic failure,” and thus implies a clinical decision and action (Reit & Kvist, 1998; Strindberg, 1956). For this reason, endodontology scholars since at least the 1980s have been puzzled and annoyed by recurrently shown variation in clinical decisions about root‐filled teeth with AP, and particularly by practitioners’ reluctance to suggest and institute an endodontic retreatment procedure (Kvist et al., 1994; Reit et al., 1985; Taha et al., 2019). The many complex factors involved in the clinical decision‐making process have made it difficult to present a coherent model for explaining and understanding these variations (Kvist, 2018).

However, there is good reason to assume that these variations can be attributed to two main categories of uncertainty: facts and values (Kvist & Reit, 2002). With regard to facts, solid scientific evidence is lacking on questions regarding both the diagnosis of a “failure” and the outcome of retreatment or a no‐intervention alternative (Frisk & Kvist, 2018). With regard to values, the variations in question may stem from different perceptions of disease, educational contexts, and values concerning illness and health (Kvist & Reit, 2002).

Due to the great uncertainties, authors have emphasized the importance of the patient's right to autonomy and hence participation in the process involving decisions on retreatment (Azarpazhooh et al., 2014; Kvist & Hofmann, 2022; Kvist & Reit, 2002).

Autonomy, or self‐determination, means that an individual has the right to decide on matters regarding his or her own body, mind, and life. The right to autonomy has a strong foundation in various ethical theories (Beauchamp & Childress, 2019). As the concept of autonomy also includes an individual's right to decide on his or her healthcare, any two‐way communication process involving information sharing and decision‐making should always precede a medical or dental decision on treatment or refraining from it (World Health Organization, 1978).

For a patient to be able to make an autonomous decision, the dentist must therefore provide the patient with all relevant facts: the findings, the etiology of the disorder, the various options available for dealing with it, and the risks, costs, probable outcome, and long‐term prognosis (Kvist, 2018; Kvist & Hofmann, 2022). Like in many other clinical situations, in the case of a root‐filled tooth with AP, many of the facts that are required for the provision of valid evidence‐based information are missing or highly uncertain (Frisk & Kvist, 2018; Kvist & Hofmann, 2022).

There is also the matter of how the available information should be presented. A choice between options can be framed in different ways. The framing effect, which was first recognized by Tversky and Kahnemann in 1981 (Tversky & Kahneman, 1981), is described as a cognitive bias whereby people decide on options on the basis of whether they—the options—are presented with positive or negative connotations.

Although this cognitive bias effect has been explored in several medical decision‐making contexts (Gong et al., 2013), its relevance has been met with very little interest among clinical researchers in dentistry (Arora, 2000). But in one study by Foster & Harrison (2008), first‐year dental students simulated the role of patients in an experiment on the effect of framing in an endodontic decision‐making situation (Foster & Harrison, 2008). In a scenario involving a symptomatic tooth with failed endodontic therapy, they were asked to select one of two treatment options: nonsurgical endodontic retreatment, or extraction and implant placement. Their selection of treatment was significantly influenced by biased presentations.

The present study was set up to explore the possible influence of a framing effect when an individual is asked to choose between no intervention and retreatment of a root‐filled tooth presenting with asymptomatic AP.

## 2 MATERIAL AND METHODS

### 2.1 Participants

A total of 248 individuals (74 men and 173 women) who studied or worked within the area of dentistry were recruited on a voluntary basis. This number included 121 dental students, all of whom were studying at the Institute of Odontology at Sahlgrenska Academy, University of Gothenburg, Sweden. They had reached various training levels, with 49 in the first year, 29 in the second, and 43 in the third. Seventy‐four participants were drawn from the staff at the Institute of Odontology: 32 dentists, 7 dental hygienists, 32 dental nurses, and 2 people in administration and reception. The 53 remaining participants consisted of general dentists, both private and public employees, who were attending a course in endodontics at the Gothenburg Dental Society.

### 2.2 Questionnaire

Two variants of a questionnaire were created, each designed to cause a respondent to answer from his or her perspective as a potential patient. The description of the clinical decision‐making situation was simple and patient‐oriented.

Although the clinical situation and information were identical in both questionnaires, the two alternative treatment options were systematically framed in two different ways.

The clinical situation was described as follows:

*Imagine that 5 years ago you were involved in a bicycle accident in which you injured your upper left central tooth. As a result, the tooth needed root canal treatment. It was opened up, cleaned of bacteria, and filled with a rubber‐like material. Finally, it was sealed with a plastic filling. Since then, you have not experienced any problems with this tooth*.

*When you come to your dentist for your annual routine checkup today, the dentist decides to take a radiograph of the tooth. This shows a lesion in the bone around the tip of the tooth*.

*On the radiograph, you can see that the bone around the root tip of the tooth is a little darker. This indicates that there are bacteria left inside the root canal that cause inflammation, which in turn becomes visible on the radiograph. The root filling appears to be short and incomplete*.

*Your dentist presents two options. You have free dental care, so any treatment you choose is free of charge*.

In the first variant of the questionnaire, the intention was to frame the options in favor of refraining from retreatment now and of waiting and seeing (FW).

*Option A (Wait)*.

*Refrain from retreatment now and wait and see. The chance that the tooth will be asymptomatic for the rest of your life is approximately 90%. The chance that any remaining infection will have no negative effect on your overall health is more than 99%*.

*Option B (Retreat)*.

*Retreatment, which involves remaking the root‐canal treatment so that the root filling becomes dense and is the correct length. Despite retreatment, the risk that the inflammation will not heal is approximately 25%*.

In the second variant of the questionnaire, the intention was to frame the options in favor of retreatment (FR).

*Option A (Wait)*.

*Refrain from retreatment now and wait and see. The risk that the tooth later will become symptomatic in the form of pain and/or swelling that requires treatment is approximately 10%. The risk that any remaining infection has a negative effect on your overall health is less than 1%*.

*Option B (Retreat)*.

*Retreatment, which involves remaking the root‐canal treatment so that the root filling becomes dense and is the correct length. The chance that the inflammation will heal is approximately 75%*.

At the end of the questionnaire, respondents were requested to register their gender, age, and occupation, or, if they were dental students, to state their year of study.

The two variants of the questionnaire are presented in Figure 1a,b.

Figure 1(a and b) The two different versions of the questionnaire (a = favoring wait and see [FW] and b = favoring retreatment [FR]) distributed to the participants in the study.

**Figure:**
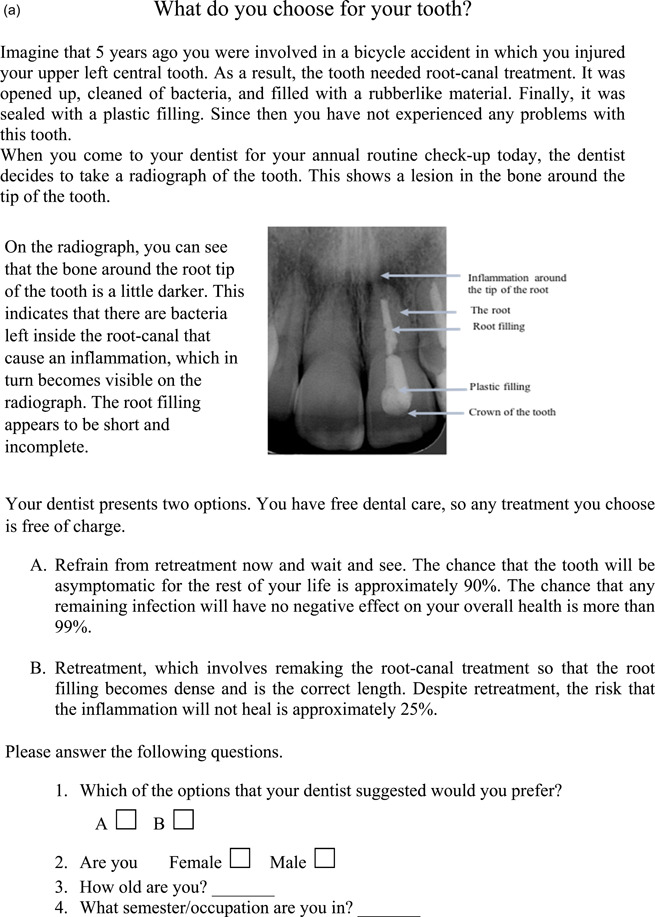

**Figure:**
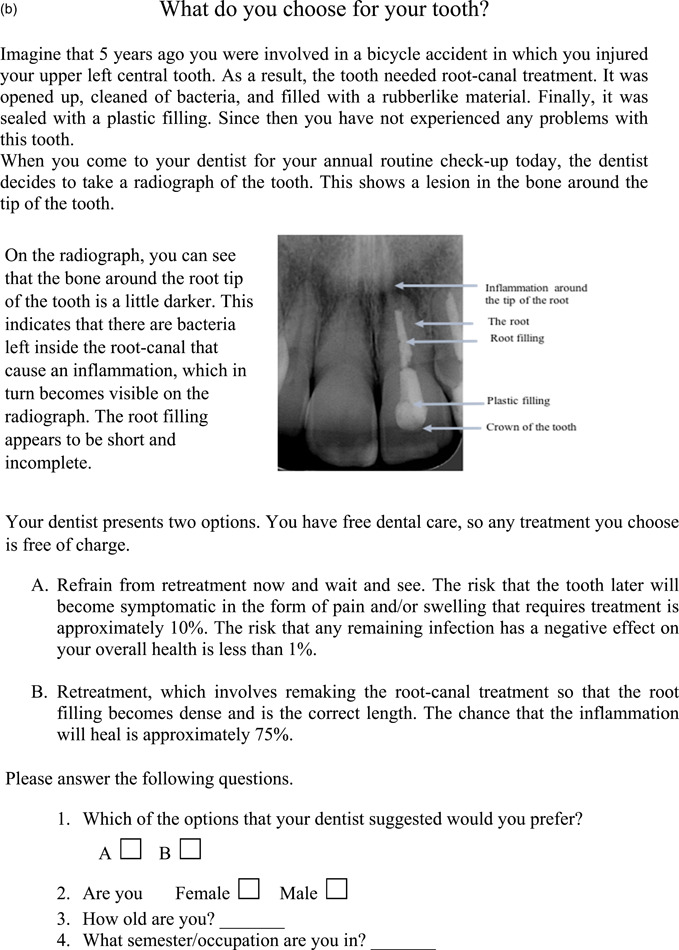

### 2.3 Distribution and procedures

The same number of copies (150) of questionnaire variants FW and FR were printed and sorted into a stack in which FW consistently alternated with FR. The questionnaires were distributed on five different occasions.

First, two of the authors (Agnesa Smakiqi and Daniela Henelius) gave a short introduction in which the participants were told, that the questionnaire was supposed to provide the basis for a Master's thesis on clinical decision‐making in root‐filled teeth. Participants were also informed that reading and answering would require no more than 10 min, that all answers would be anonymous, and that as participation was completely voluntary, the questionnaire could also be returned unanswered. Participants were asked not to communicate with each other when completing the questionnaire.

The real purpose of the study was concealed from the participants, as was the fact that two different variants of the questionnaire would be distributed. The stack of questionnaires was distributed, with each participant receiving only one questionnaire.

The questionnaire was distributed to the students in the lecture hall during a selected lecture and to the staff at the Folktandvården Education Clinic for Dentistry during a staff meeting. These questionnaires were all collected immediately. Distribution of the questionnaire among dentists took place during an evening course organized by Gothenburg Dental Association (GTS). These participants were required to submit the questionnaire either immediately, or by post in a prestamped letter to the Department of Endodontology. The information from each questionnaire was then transferred to an Excel data sheet (Microsoft Corp).

### 2.4 Ethical considerations

This study was originally a part of a master's thesis at the Institute of Odontology, Sahlgrenska Academy, University of Gothenburg, Sweden. No patient or patient data was involved in the study, except for an anonymous radiograph, of which the patient had given consent to be used for the purpose. All responders to the questionnaire were informed that answering would be anonymous, and that participation was completely voluntary, the questionnaire could be returned unanswered without registration.

### 2.5 Statistical methods

Before the statistical analysis, participants were divided by gender and into three groups on the basis of age: 18–25, 26–49, and 50+. We also categorized the respondents as follows: students and of their training level (1st, 2nd, or 3rd year), staff at dental school or course for dentists. For comparison between the different groups, Fisher's exact test was used with a two‐sided 5% significance level found at https://www.graphpad.com.

## 3 RESULTS

A total of 248 individuals participated in our study, 141 of whom (56.9%) chose retreatment and 107 of whom (43.1%) chose the wait‐and‐see option.

One hundred and twenty‐five participants (50.4%) had received the questionnaire variant framed in favor of refraining from treatment (wait‐and‐see) (FW), and 123 participants (49.6%) had received the variant framed in favor of retreatment (FR). Whereas 69 (55.2%) of the participants who had received questionnaire FW chose the option to refrain and wait, 56 (44.8%) chose retreatment. In contrast, whereas 38 (30.9%) of the participants who had received questionnaire FR chose to refrain and wait, 85 (69.1%) chose retreatment. This difference was statistically significant (*p* = .0002) (Table 1.)

**Table 1:** Choices of the wait and see or the retreatment option in relation to which questionnaire variant responding to favoring wait and see (FW), favoring retreatment (FR).

	Questionnaire FW		Questionnaire FR		
	(*n* = 125)		(*n* = 123)		
Variables choice	*n*	%	*n*	%	*p*‐value
All participants					
Wait and see	69	55.2	38	30.9	
Retreatment	56	44.8	85	69.1	.0002
Gender					
Male					
Wait and see	17	51.5	14	34.1	
Retreatment	16	48.5	27	65.9	.20
Female					
Wait and see	52	57.1	24	29.3	
Retreatment	39	42.9	58	70.7	.0004
Age					
18–25 years old					
Wait and see	36	66.7	23	42.6	
Retreatment	18	33.3	31	57.4	.020
26–49 years old					
Wait and see	14	50.0	3	17.6	
Retreatment	14	50.0	14	82.4	.060
50+ years old					
Wait and see	18	43.9	12	25.0	
Retreatment	23	56.1	36	75.0	.098
Category/Occasion					
1st year students					
Wait and see	16	64.0	9	37.5	
Retreatment	9	36.0	15	62.5	.12
2nd year students					
Wait and see	7	46.7	6	42.9	
Retreatment	8	53.3	8	57.1	1.00
3rd year students					
Wait and see	19	86.4	10	47.6	
Retreatment	3	13.6	11	52,4	.016
Staff at dental school					
Wait and see	18	46.2	9	25.7	
Retreatment	21	53.8	26	74.3	.11
Course for dentists					
Wait and see	9	37.5	4	13.8	
Retreatment	15	62.5	25	86.2	.093

*Note*: For categorical variables *n* (%) is presented. For comparison between groups Fisher's Exact test (lowest one‐sided *p*‐value multiplied by 2) was used for dichotomous variables. Data on gender were missing for one participant and on age for six individuals.

Seventy‐four (30%) of the participants were men and 173 (70%) were women. When the possible framing effect was analyzed on the basis of gender, a statistically significant framing effect (*p* = .0004) was found among women. In men, a framing effect was registered numerically, but the difference was not statistically significant (*p* = .20) (Table 1).

One hundred and eight participants (45%) were in the 18–25 age group; 45 (18%) in the 26–49 age group; and 89 (37%) in the 50+ age group. A statistically significant framing effect (*p* = .020) was detected in the 18–25 age group. In the 26–49 and 50+ age groups, the framing effect was not statistically significant (Table 1). The results were also analyzed on the basis both of occupational category and of the occasion on which the questionnaire was distributed. A framing effect was observed regardless of the category but reached statistical significance only among 3rd year students (*p* = .016) (Table 1).

## 4 DISCUSSION

The results of this study show that a framing effect can be expected to play a role in endodontic retreatment decision‐making. The pooled data from all respondents showed a statistically significant effect. A similar effect was seen when the respondents were divided into subgroups categorized by gender, age, occupation, and occasion. However, the effect was not statistically significant in all analyses. Even if it cannot be ruled out that in some groups of respondents, no framing effect is present, the probable reason is that some of the subgroups were not large enough, thereby causing a statistical Type II error. Future research projects could aim to evaluate whether the framing effect is greater, less, or not at all demonstrable in different groups of potential decision‐makers depending on gender, age, level of education, or other group‐defining characteristics.

The explanation for the framing effect can be found within the framework of the prospect theory (Kahneman & Tversky, 1979). This theory, which essentially concerns economic behavior, challenged the idea of rationality among decision‐makers as it had explicitly been formulated in the expected utility theory (Von Neumann & Morgenstern, 1947).

The prospect theory, which was based on results from controlled studies, describes how individuals assess their loss and gain perspectives in an asymmetric manner. The theory assumes that there are two different phases of decision‐making. In the first phase, the alternatives stated are automatically evaluated, a process that involves analysis and simplification of the information they contain. In the second phase, the decision‐maker considers the alternatives and chooses the one he or she judges to be most beneficial (Kahneman & Tversky, 1979). When a choice is being made between two options, an alternative that is described in its entirety positively seems to be preferable to one that is in itself described negatively, even though both alternative descriptions state exactly the same factual information.

In a classic study by McNeil et al. (1982), it was investigated how variations in the way in which information was presented influenced the choices made by ambulatory patients, graduate students, and physicians when deciding between alternative therapies—radiation or surgery—in cases of lung cancer. Different groups of respondents received input data that differed according to whether the treatment outcomes were framed in terms of the probability of living or the probability of dying. In all three groups of responders, the attractiveness of surgery relative to radiation therapy was greater when the problem was framed in terms of the probability of living rather than in terms of the probability of dying.

In our study involving the root‐filled tooth with AP, the factually identical information on the “wait and see” and “retreatment” options were framed either by using the word “chance” or the word “risk” to indicate the probability (likelihood) of outcomes. However, the connotations of the words “risk” and “chance” are essentially different (Li et al., 2020; Morizot, 2012). While chance has a positive connotation (the likelihood of something good happening), risk has a negative connotation (the likelihood of something bad happening). By using a more neutral word such as “probability” or “likelihood,” a clinician presenting prognostic assumptions about a clinical option could possibly reduce the framing effect.

To enhance the framing effect in our experiment, we combined the word “chance” with “healing” (a positively laden expression), and “risk” with “nonhealing” (a negatively laden expression).

Similarly, a statement of a 90% likelihood of success highlights the attractive outcome of a procedure, whereas a 10% likelihood of failure tends to highlight the unattractive outcome. Partly because of the strongly value‐laden component of the “success” and “failure” classifications, various authors have suggested alternative systems and terms to evaluate and classify the outcome of root canal treatment (Friedman & Mor, 2004; Messer & Yu, 2013; Wu et al., 2011).

Language evidently plays an important role in many aspects of medicine and healthcare and may be used as a powerful tool in clinical decision‐making situations (Srivastava, 2019). Thus, the clinician presenting the information to the patient may, consciously or unconsciously, influence the patient's choice in favor of a particular option.

In the absence of strong scientific evidence for the benefits or harms of a particular choice, it may also be assumed that the clinician's framing of the options and how presenting them to the patient, is influenced by different heuristic biases concerning probability (Hicks & Kluemper, 2011; Reit et al., 1985; Tversky & Kahneman, 1974). Referred to as “availability,” defines the phenomenon whereby people assess the frequency of a class or the probability of an event on the basis of the ease with which instances or occurrences can be brought to mind (Tversky & Kahneman, 1974). For example, the influence of availability may be expected when the retreatment and wait‐and‐see options for new patients with an asymptomatic AP are framed by a dentist who recently met a patient with a flare‐up in a root‐filled tooth. In particular, this is to be expected if, in any aspect, the present case resembles the recent experience of a patient in severe pain, as explained by the principle of “representativeness” (Hicks & Kluemper, 2011; Tversky & Kahneman, 1974). Representativeness is defined as a heuristic bias that occurs when the similarity of objects or events confuses people's thinking regarding the probability of an outcome. Quite apart from the heuristic reasoning about the probability that may influence dentists’ expectations and preferences, their clinical choices may even be affected by their prejudices about their patients (Patel et al., 2019).

An interesting finding in our study was that even though a framing effect was evident at the group level, both options, regardless of the variant of the questionnaire, were chosen rather frequently. This finding indicates that other factors than how the information was framed are important for the respondent's choice. This is consistent with previous studies on the subject of endodontic retreatment decision‐making (Kvist, 2018; Reit & Kvist, 1998).

The praxis concept theory as regards endodontic retreatment decision‐making was proposed by Kvist et al. (1994). At its core is the hypothesis that interindividual variation in decision‐making on endodontic retreatment can largely be explained by variation in the values of the individual decision‐makers. The observations in this study, which involved respondents with varied experiences and backgrounds, do not falsify this theory. To explain the origin of the various values involved in the endodontic retreatment decision‐making process, it is assumed that, somehow, an individual's values are at least partly a merged mental disposition developed by experience from different environments (Kvist, 2018; Kvist & Reit, 2002; Kvist et al., 1994; Taha et al., 2019). For example, it has been shown that, in endodontic retreatment decision‐making situations, endodontists systematically make decisions differently than students, general dental practitioners, or specialists in other disciplines (Bigras et al., 2008).

Any acknowledgment of the framing effect challenges the concept of patient autonomy and informed consent. By consciously or unconsciously choosing value‐laden words and framing different treatment options, a therapist will influence their patient's choice, intentionally or otherwise. Only if the dentist is aware of this problem, and consciously attempts to provide information in ways that are as neutral as possible will he or she be able to reduce this effect.

On the other hand, the power of the framing effect in clinical decision‐making may also be deliberately used in situations to influence patients to make the “right” decision—“right” in the sense that there are good reasons and good evidence to believe that a certain decision is in the patient's best interests. Sherman et al. (2008) and Patel et al. (2019) showed how the framing effect was used to encourage the use of dental floss: those who received information in the form of a profit‐framed video were more likely to use dental floss according to the recommendations for a period of 6 months than those who saw a loss‐framed video.

This study makes no claim to fully chart the framing effect of clinical decision‐making in connection with root‐filled teeth. As the respondents were not selected on the basis of belonging to nonprofessional groups and as all had some kind of affiliation to dentistry, its external validity can be questioned. It may also be argued that the factual information—that is, the percentages regarding healing, the likelihood of becoming symptomatic, and the influence of any remaining infection and inflammation on systemic health—is not based on the best available evidence. However, it was not our purpose to systematically review the best current evidence on the matter. Instead, our purpose was to apply the phenomenon of framing to a well‐known clinical decision problem within endodontics, to provide some empirical support, and to discuss various questions that arose, and also their implications.

## 5 CONCLUSION

A framing effect is likely to play an essential role in endodontic retreatment decision‐making of root‐filled teeth with asymptomatic AP.

## AUTHOR CONTRIBUTIONS

Thomas Kvist, Daniela Henelius, and Agnesa Smakiqi all made substantial contributions to the conception and design of the study. Daniela Henelius and Agnesa Smakiqi were responsible and involved in data collection. Thomas Kvist, Daniela Henelius, and Agnesa Smakiqi were all involved in data interpretation, statistical analyses, drafting, and critically revising the manuscript. All authors have given final approval for the version to be published.

## CONFLICT OF INTEREST STATEMENT

The authors declare no conflict of interest.

## Data Availability

Data that support the findings of this study are available from the corresponding author upon reasonable request.
